# Adventures in Billing: Sustainable Billing Practices in Serving Adults With Complex Health Needs

**DOI:** 10.1093/geroni/igaf122.320

**Published:** 2025-12-31

**Authors:** Maresi Berry-Stoelzle, Sarah Murray, Tammy Walkner, Daniel Karr, Amanda Heeren

**Affiliations:** Iowa City VA Health Care System, Iowa City, Iowa, United States; Iowa City VA Medical Center, Iowa City, Iowa, United States; Iowa City VA Health Care System, Iowa City, Iowa, United States; Department of Veterans Affairs, Iowa City, Iowa, United States; Iowa City VA Health Care System, Iowa City, Iowa, United States

## Abstract

Discussions about future medical care are first steps to life altering medical decisions. A complex medical diagnosis with accompanying therapy affects the patient and their family/support network. Communication between patients, family members, and the medical care team can lower anxiety, increase goal congruent care, and increase satisfaction in care received. This is grouped together as advance care planning (ACP). The Centers for Medicare and Medicaid Services (CMS) has added a billing code, available for all patients and licensed independent providers to have dedicated medical encounters focused on ACP. Unfortunately, integration of these encounters into routine outpatient medical care has been low. For a rural serving Veterans Health Administration (VHA) Medical Center, <1% of eligible Veterans were billed using these codes. In a more generalized study, 5.2% of eligible VHA patients had documentation of ACP in 2020. Especially rural Veterans and others with extended travel time may have challenges adding another encounter to an already busy medical care schedule. Our VHA initiative has built and tested a concise, billable telehealth ACP encounter to integrate care between VHA specialty clinics and long-term care facilities where Veterans receive care. By adhering to the CPT code guidelines and integrating practices from VHA palliative care and ethics trainings, we are able to offer Veteran-centered encounters to patients and families. Significant benefits accrue for Veterans, families, and the health care system in ensuring that patient records and Veteran/family expectations are regularly updated. We discuss offering this encounter to almost 1,000 Veterans and families.

